# Lower Island Trapezius Flap for a Rare Scalp Tumor

**DOI:** 10.7759/cureus.72903

**Published:** 2024-11-02

**Authors:** Aravind Ramkumar

**Affiliations:** 1 General Surgery, Malavya Hospital, Bengaluru, IND; 2 General Surgery, Employees State Insurance Corporation (ESIC) Medical College, Post-graduate Institute of Medical Sciences and Research (PGIMSR) Hospital, Bengaluru, IND

**Keywords:** dorsal scapular artery, lower island trapezius flap, proliferating trichilemmal tumor, scalp tumor, trapezius flap

## Abstract

A 54-year-old male with a past history of acute pancreatitis and pulmonary embolism presented with a scalp tumor in the occipital region for a six-month duration. With a biopsy report of squamous cell carcinoma, wide local excision with the lower island trapezius myocutaneous flap with skin grafting was done. However, the final pathology report revealed a proliferating trichilemmal tumor with clear margins. The technique of the lower island trapezius flap in posterior scalp defects and a brief overview of proliferating trichilemmal tumors are described in this report.

## Introduction

The majority of scalp tumors are benign, with only 1-2% being malignant [1]. Non-melanoma skin cancers, including squamous cell carcinoma and basal cell carcinoma, are the most common malignant tumors. Reconstruction following resection of scalp tumors could be primary closure, local flaps like Orticochea flap, regional flaps like temporo-parietal-occipital (Juri flap), trapezius myocutaneous flap, latissimus dorsi myocutaneous flap or free tissue transfer depending on the location, size, and thickness of defect, and other factors [2,3]. Postero-lateral scalp defects can be reconstructed with a lower island trapezius flap [4], and understanding the vascular anatomy is key to the success of the surgery.

## Case presentation

A 54-year-old male presented with scalp swelling with ulceration in the occipital and retroauricular regions on the right side for a six-month duration. He had a history of previous admission for acute pancreatitis and pulmonary embolism managed conservatively. A biopsy done under local anesthesia was suggestive of moderately differentiated squamous cell carcinoma.

A contrast CT of the brain, neck, chest, and abdomen was done, and there were no distant metastases, no destruction of the underlying bone, and no significant regional lymphadenopathy (Video 1).

**Video 1:** CT of the brain A scalp tumor is seen without underlying bone destruction.

**Figure 1:**
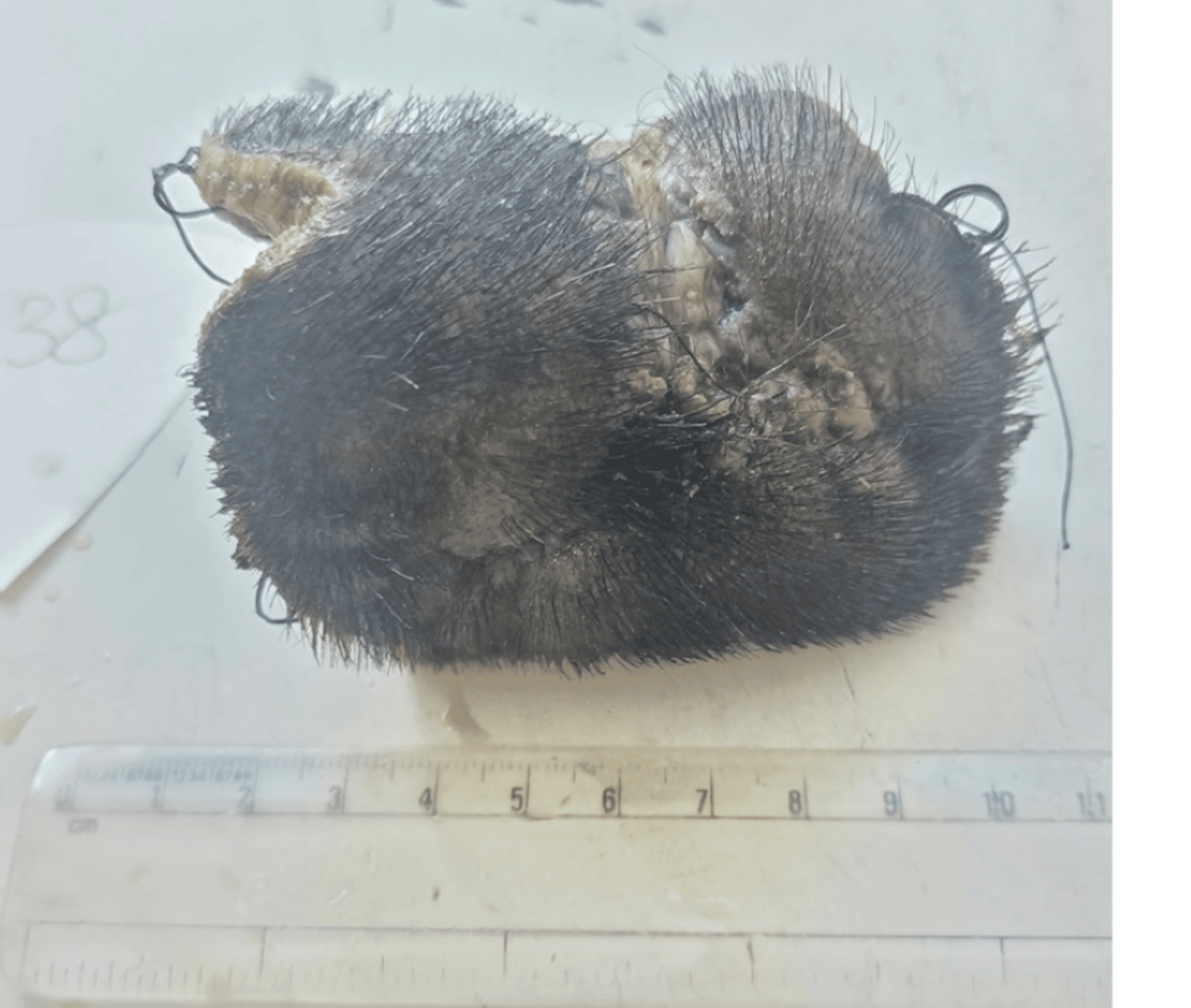
Gross appearance of the tumor in the occipital region of the scalp

Under general anesthesia with a lateral position, a wide local excision of the tumor with a 1 cm gross margin was done with resection of the pericranium in the region beneath the tumor. In the “margin area,” where there was no gross tumor, the pericranium was spared. The defect size was 9 × 9 cm (Figure 1).

A decision was reached, and a lower island trapezius flap was preferred over microvascular free tissue transfer, given the history of pulmonary embolism. A vertical island was fashioned with a lower limit of the flap extending beyond 5 cm below the angle of the scapula with the idea that if the distal part is non-viable, it could be excised at the time of inset. The myocutaneous flap was raised to approximately the spine of the scapula. The pedicle could be visualized on the deep surface of the flap. The dorsal scapular artery (DSA) supply to the flap was preserved since it has been said that for the lower island trapezius flap to be reliable, the DSA supply may preferably be preserved [4]. The skin over the posterior aspect of the neck was incised, leading up to the defect, and undermined on both sides to accommodate the flap, which was turned to inset on the defect. The flap covered most of the defect except a small portion in the retroauricular region, where the pericranium was intact, and a split skin graft was used to surface that defect. 

**Figure 2:**
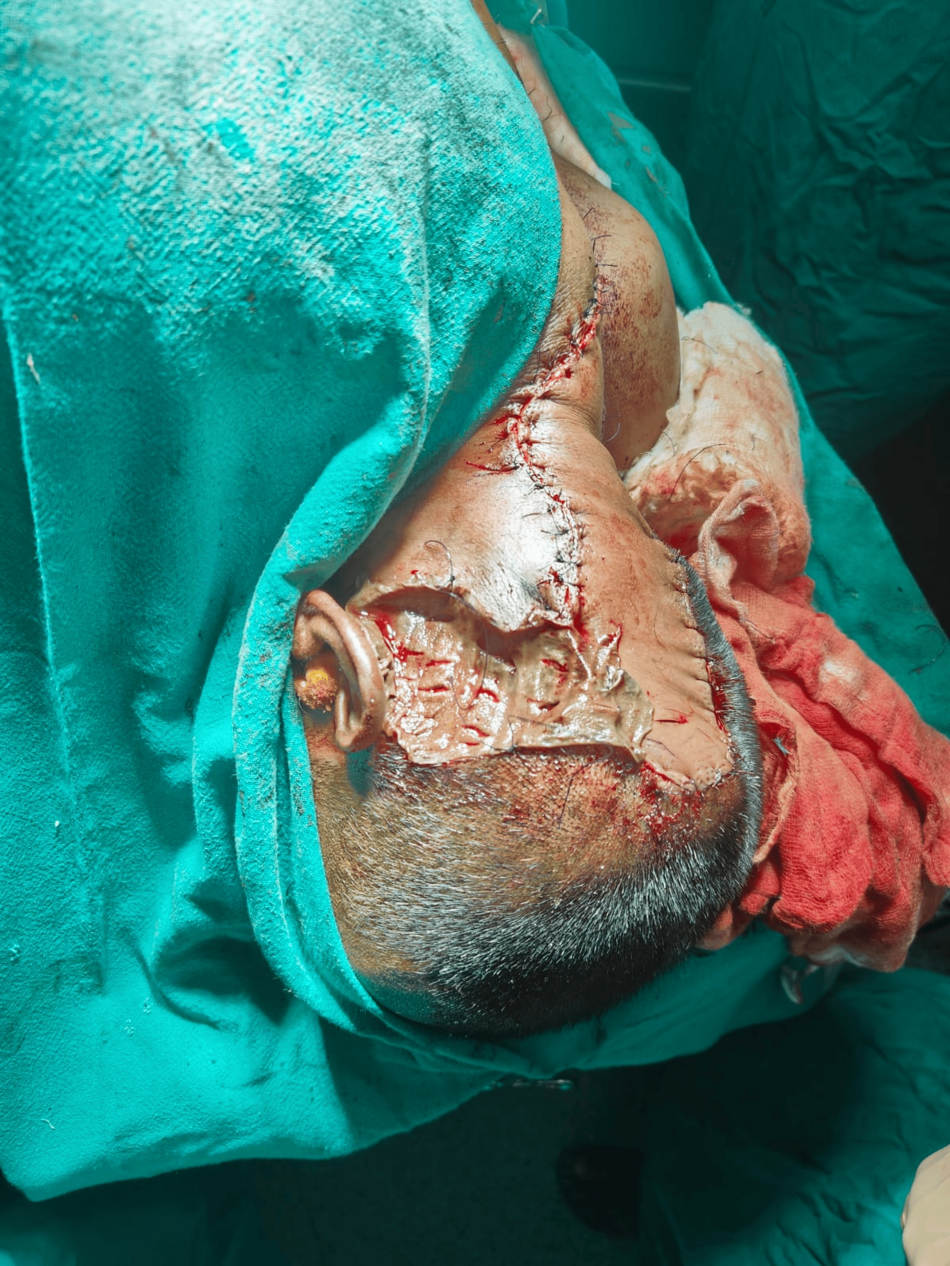
Intraoperative view A flap inset, along with a skin graft adjacent to the flap, is seen.

**Figure 3:**
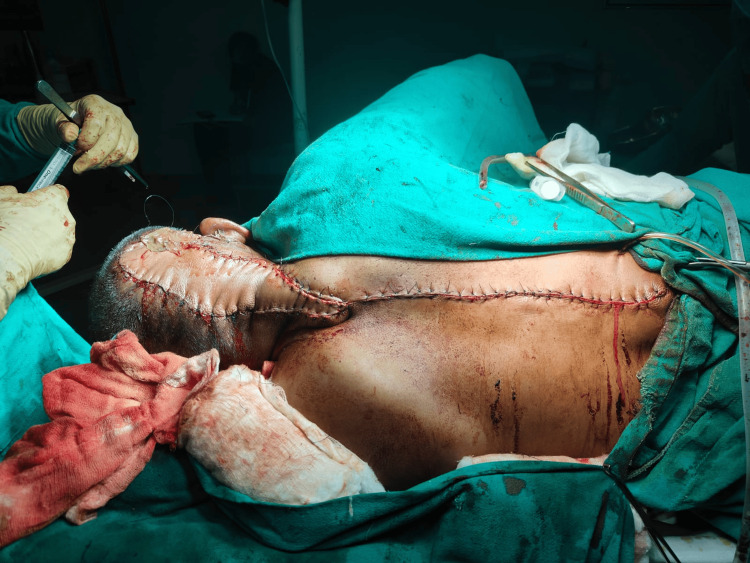
Intraoperative view Image showing a flap inset into the scalp defect and skin graft in the right retro-auricular region adjacent to the flap and primary closure of donor areas.

A suction drain was placed in the donor area, and primary closure of the donor area was done (Figures 2, 3).

**Figure 4:**
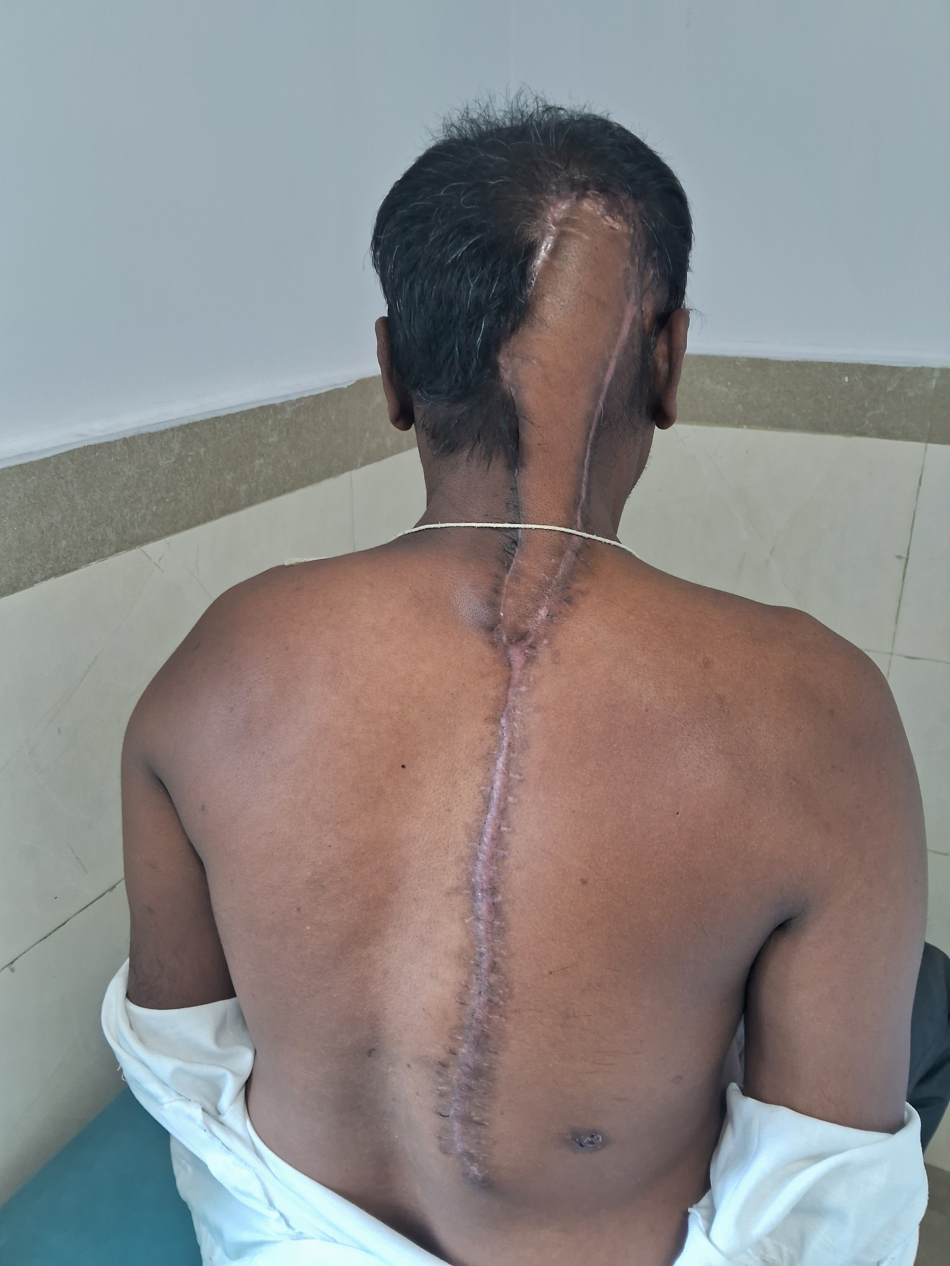
Postoperative view The healed donor area and flap are shown.

**Figure 5:**
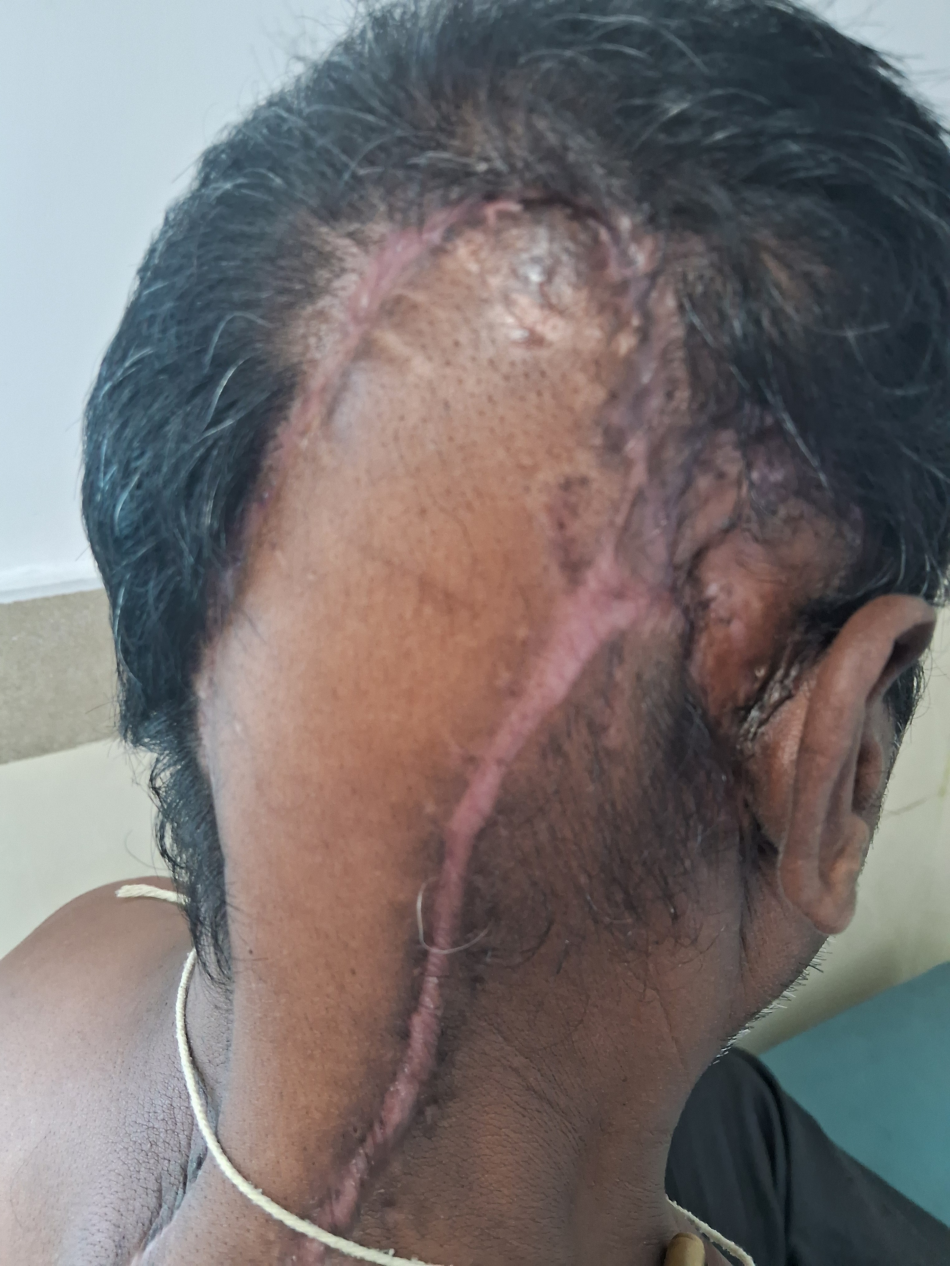
Postoperative view The postoperative appearance of the flap and skin graft is seen.

The flap and split skin graft survived entirely (Figures 4, 5), though there was transient venous congestion in the tip of the flap.

**Figure 6:**
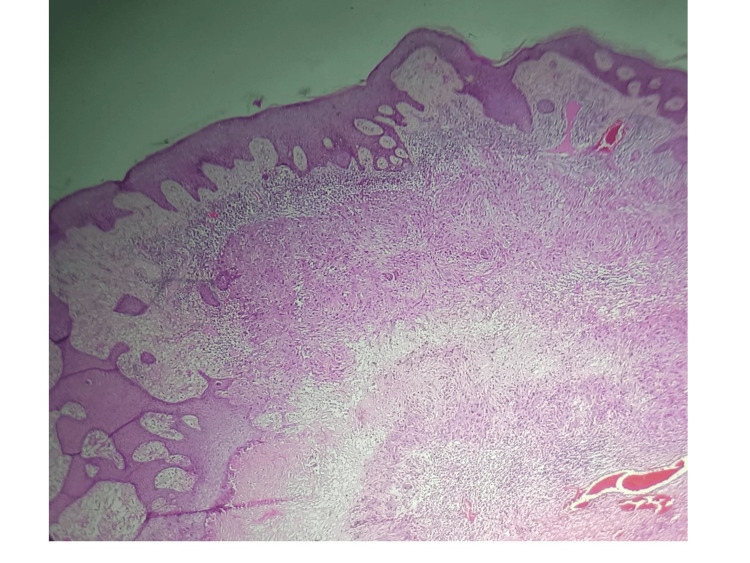
Hematoxylin & eosin (H&E) staining with 10× magnification The upper dermis shows a partially circumscribed neoplasm composed of sheets of proliferating squamous cells.

**Figure 7:**
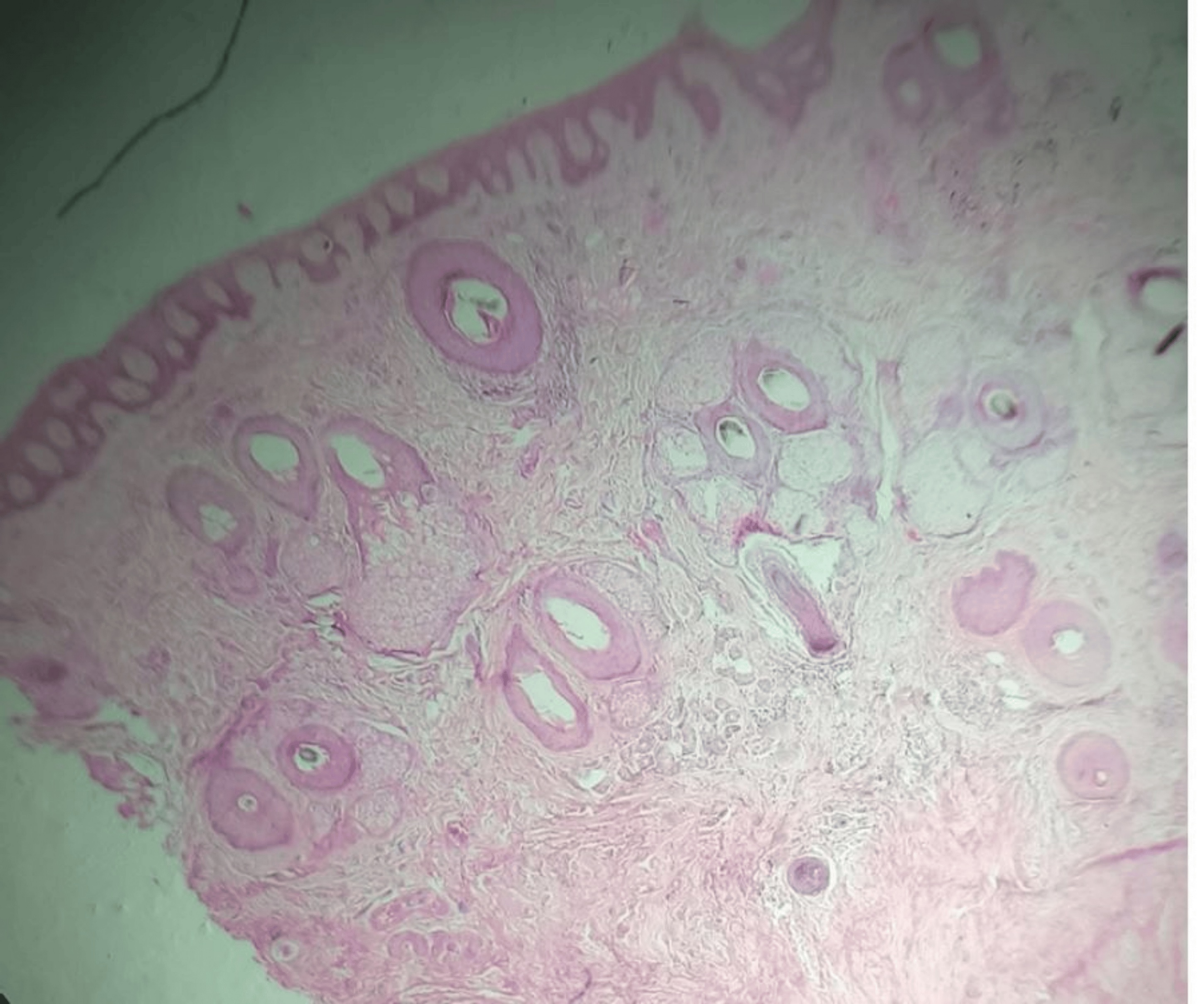
Hematoxylin & eosin (H&E) staining with 10× magnification Numerous hair follicles and adnexal structures are noted.

**Figure 8:**
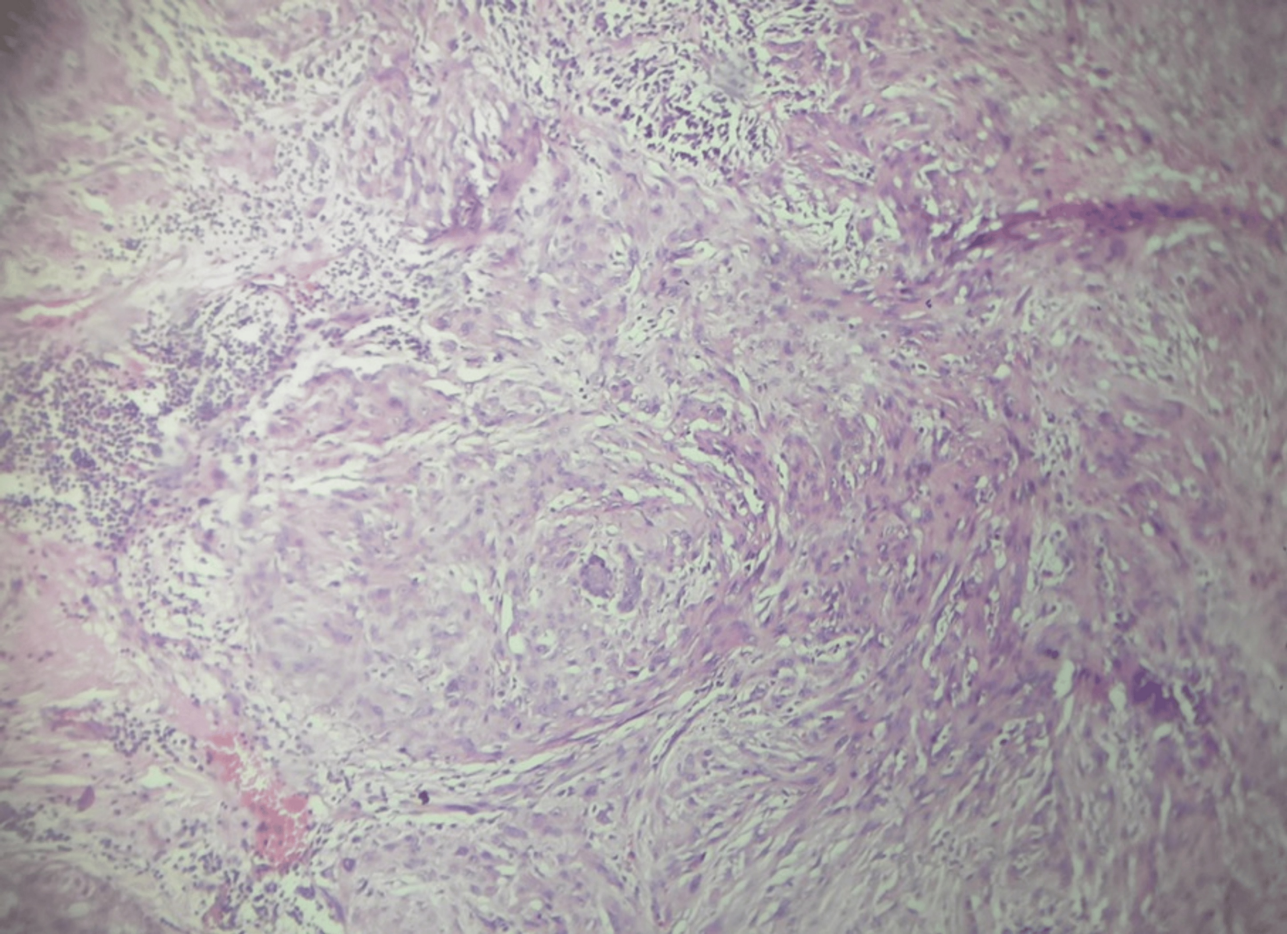
Hematoxylin & eosin (H&E) staining with 40× magnification Individual tumor cells show moderate pleomorphism and occasional mitotic figures.

The final pathology report revealed a proliferating trichilemmal tumor with circumferential and deep margins free of tumor (Figures 6-8).

## Discussion

The trapezius flap has been used for the reconstruction of posterior scalp defects [2,4,5]. It has been advised that the vertical skin paddle does not exceed 5 cm below the inferior angle of the scapula for the lower island trapezius myocutaneous flap [4,5]. In contrast, others have opined that it can be extended to 15 cm below the inferior angle of the scapula if the DSA is preserved, while the lower limit of the skin island should be within the inferior angle of the scapula if the DSA is divided [6]. The vascular anatomy and surgical technique to be adapted to the vascular anatomy have been described in a cadaveric study [6]. In this study, it has been shown that two blood vessels, namely the transverse cervical artery (TCA) and DSA, contribute to the vascular supply of the lower island trapezius myocutaneous flap. The TCA branches off the thyrocervical trunk, passes over the brachial plexus and levator scapulae, and descends down into the investing fascia on the deep surface of the trapezius. The DSA branches off the subclavian artery lateral to the scalenus anterior, 2 cm lateral to the thyrocervical trunk, passes between the branches of the brachial plexus, and then deep to the levator scapulae. At the level of the spine of the scapula, it divides into two branches: the larger one passing between the rhomboid minor and major and continuing deep to the trapezius and the smaller branch passing behind the rhomboid major and giving off muscular branches. In their study, the authors found that, in most of the cadavers, either TCA or DSA was dominant. When either was dominant, the proximal portion (proximal to the levator scapulae) of the non-dominant vessel did not exist, but the distal portion (distal to the levator scapulae) followed the constant anatomy described above. When both vessels were dominant, they had both separate and independent origins.

Based on this study, we followed the technique described in the technical report section. We created a vertical skin island extending beyond the inferior angle of the scapula wherein the distal portion of the flap was devoid of the underlying trapezius muscle and raised the myocutaneous flap, preserving the TCA supply. When we reached the point of entry of the DSA between the rhomboids, we preserved the DSA branch to the flap, and we had sufficient flap length to reach the defect, based on the preoperative contrast CT where we could see the prominent DSA supply to the flap (Video 2).

**Video 2:** Video showing the course of DSA Arrow points to the course of DSA, and circle highlights the entry of DSA between the rhomboid major and minor into the deep aspect of the trapezius muscle. DSA, dorsal scapular artery

Proliferating trichilemmal or pilar tumor arises from the outer root sheath of the hair follicle in the dermis or subcutaneous. It is more common in women and occurs on the scalp. They have been grouped into three types: benign, locally aggressive, and potential for metastasis based on growth pattern, nuclear atypia, mitosis, necrosis, and perineural and vascular invasion [7]. Surgical excision with clear margins is the recommended treatment [8], which was achieved in our patient. Since it is a rare tumor, there is no clear guideline on the use of adjuvant radiotherapy. We decided to follow up with the patient regularly since the margins of the resection were free of tumors.

## Conclusions

To conclude, a proliferating trichilemmal tumor is an uncommon tumor of the scalp and can be mistaken for squamous cell carcinoma. Its behavior depends on the histopathological features of the tumor. The lower island trapezius myocutaneous flap can be based on the transverse cervical with DSA with reach to the ipsilateral occipital region comfortably.
